# Exploring the Sustainability of Upcycled Foods: An Analysis of Consumer Behavior in Taiwan

**DOI:** 10.3390/nu16152501

**Published:** 2024-07-31

**Authors:** Min-Yen Chang, Kung-Ling Lai, I-Kai Lin, Ching-Tzu Chao, Han-Shen Chen

**Affiliations:** 1 Department of Accounting, Jiaxing University, Jiaxing 314001, China; mingyen0223@zjxu.edu.cn; 2 In-Service Master Program of International Health Industry Management of College, Chung Shan Medical University, Taichung 40201, Taiwan; ds178294@gmail.com; 3 Department of Health Industry Technology Management, Chung Shan Medical University, Taichung 40201, Taiwan; linken910428@gmail.com (I.-K.L.); tiffany891224@gmail.com (C.-T.C.); 4 Department of Medical Management, Chung Shan Medical University Hospital, Taichung 40201, Taiwan

**Keywords:** upcycled food, food choices, food waste, food safety, Sustainable Development Goals (SDGs)

## Abstract

Given the urgent climate change and food security challenges, upcycled food products are crucial for sustainable food production and waste management. This study investigates Taiwanese consumer behavior towards upcycled foods using the value–attitude–behavior (VAB) theory, focusing on “product knowledge”, “green perceived quality”, and “price sensitivity”. Of the 335 distributed surveys, 320 valid responses (95.5% effectiveness) were analyzed. The results indicated that eco-conscious values strongly influenced consumer attitudes and anticipated guilt (β = 0.647, *p* < 0.001; β = 0.691, *p* < 0.001), shaping behavioral intentions (β = 0.290, *p* < 0.001). Attitudes significantly correlated with intentions, validating the VAB framework. However, anticipated guilt showed a minimal impact (β = 0.029, *p* = 0.629), revealing complex consumer emotions. Green perceived quality and product knowledge were the key decision-making factors (β = 0.193, *p* < 0.001; β = 0.146, *p* < 0.001). Surprisingly, price sensitivity positively influences intentions (β = 0.764, *p* < 0.001), suggesting the consumer prioritization of quality and environmental values over price. These insights inform strategies for businesses to enhance consumer engagement and sustainability alignment, advancing progress towards Sustainable Development Goals (SDGs).

## 1. Introduction

Amidst the pressing global challenges of profound climate change and food security, greenhouse gas (GHG) emissions stemming from food production and waste management have emerged as critical issues that require immediate attention. Garske et al. [1] and Rodriguez Garcia and Raghavan [2] highlighted the substantial impact of this dilemma on climate change and food security.

According to the Food and Agriculture Organization of the United Nations (FAO) [3], approximately one-third of the world’s food supply is wasted annually during production and consumption, resulting in 8–10% of the total greenhouse gas emissions, while an estimated 800 million people worldwide grapple with hunger. In the pursuit of Sustainable Development Goals (SDGs), particularly targeting “ending hunger” (SDG2) and achieving “responsible consumption and production” (SDG12), the concept of upcycling and repurposing food has emerged as a pivotal element in realizing these aspirations [4,5].

The Upcycled Food Association (UFA) [6] defines upcycled food as products primarily utilizing ingredients that would otherwise go to waste, boasting a transparent supply chain and positive environmental impacts. A growing body of research has investigated the demand for upcycled foods and their ecological and societal implications. Coderoni and Perito [7], Thorsen et al. [8], and Grasso et al. [9] indicated that consumers favor these products because of the dual benefits of waste reduction and the preservation of nutritional content. Nogueira et al. [10] highlighted the nutritional benefits of upcycled foods, thereby enriching the diets of low-income households.

In the realm of fostering a circular economy, Peschel and Aschemann-Witzel [11] highlight the positive economic role of repurposing solid waste in agricultural food sectors. For instance, in 2023, Salt and Straw, an American ice cream brand, collaborated with multiple organizations to release five upcycled ice cream flavors, estimated to save 38,000 pounds (approximately 17,000 kg) of food waste annually. Companies such as the Renewal Mill have committed to reusing byproducts from plant milk production, creating flour, and baking products to curb the environmental impacts of food waste. With increasing environmental consciousness among consumers, their inclination towards upcycled products has grown, as supported by Perito et al. [12], Bhatt et al. [13], and Asioli and Grasso [14]. Research by Goodman-Smith et al. [15] reinforces that food transformation into new products effectively combats waste while enhancing consumer acceptance of upcycled foods.

Despite the evident advantages of upcycled foods, the academic exploration of consumer purchasing intentions remains limited. Existing research on upcycled foods primarily focuses on consumer perceptions, acceptance, and purchasing behavior. Goodman-Smith et al. [15] found that consumers generally hold positive attitudes towards upcycled foods, but their understanding of the specific benefits and manufacturing processes is limited. Bhatt et al. [16] emphasized the importance of upcycled food labeling, while Stelick et al. [17] highlighted the impact of sustainability information and nutritional content on consumer purchase intentions.

However, these studies are predominantly based on Western markets, with limited research on consumer behavior in other regions, particularly Asia. Additionally, research on the role of price sensitivity and product knowledge in purchasing decisions related to upcycled foods remains scarce.

This study aims to address these research gaps by investigating Taiwanese consumers’ attitudes, purchase intentions, price sensitivity, and product knowledge of upcycled foods, providing new perspectives and contributions to the existing literature.

The value–attitude–behavior (VAB) model, introduced by Homer and Kahle [18], serves as a common framework for understanding consumer behavior. The VAB model posits that values influence behavior through attitudes, a concept with enduring relevance and practical application. Prior studies by Issock et al. [19], Kim and Hall [20], Cheung and To [21], Ma and Chang [22], and Kim et al. [23] efficiently utilized this model to probe sustainable consumption behaviors, highlighting its predictive capabilities for consumer behavior. This study delves into its empirical application in sustainable consumption, focusing on upcycled food. The foundational notion that abstract values can shape individual actions via attitude formation, as articulated by Homer and Kahle [18], resonates with contemporary research by scholars, such as Issock et al. [19], Kim and Hall [20], Chang et al. [24], Lee et al. [25], and Wang et al. [26], exemplifying the versatility of the VAB model across various consumption contexts.

Scholarly investigations by Szakos et al. [27] emphasize the dual dimensions of emotion and cognition in attitude formation, a concept bolstered by Habib et al.’s [28] findings. Furthermore, research by Lu et al. [29] illuminates the pivotal role of anticipated guilt, a significant emotional state, as a mediating factor between values and behavioral intentions. Complementing this, Haws et al.’s [30] seminal work on delineating green consumption values provides a robust theoretical underpinning for analyzing green consumption behavior. Building on this foundation, hypotheses H1a and H1b provide insights into how consumers’ attitudes towards upcycled food and anticipated guilt stem from their green consumption values.

By examining how attitudes towards upgraded recycled foods and anticipated guilt shape behavioral intentions, this study referenced the findings of Deci [31], Lu et al. [29], and Zeynalova and Namazova [32], underscoring the pivotal role of emotions and intrinsic motivation in behavioral intentions. Prior research has established the association between green values, motivations for buying green products, attitudes towards such products, and the resulting willingness to purchase green products [33]. Studies indicate that anticipated guilt positively impacts the intention to engage in low-carbon consumption behaviors [34]. Thus, Hypotheses H2a and H2b aim to elucidate the positive relationships between these variables.

The pivotal role of consumer knowledge in shaping behavior, as underscored by Philippe and Ngobo [35], emphasizes the significance of product knowledge in consumer decision-making processes. Building on this foundational understanding and incorporating insights from subsequent studies by Peng et al. [36] and Ayub and Kusumadewi [37], this study posits Hypothesis H3, which elucidates the positive impact of product knowledge on behavioral intentions towards upcycled food products.

In examining green perceived quality, this study draws upon Zeithaml’s [38] definition of perceived quality and its implications for consumer behavior [39], inspired by the findings of Riva et al. [40]. This exploration underscores the affirmative association between perceived green quality and behavioral intentions, with research indicating a direct link between perceived quality and behavioral intentions of the millennial generation [41]. Thus, H4 sought to elucidate this relationship.

Finally, insights from Ogiemwonyi [42], Solomon and Panda [43], and Grasso and Asioli [44] highlight the critical role of price sensitivity in consumer decision-making processes. Research posits that individuals with lower price sensitivity exhibit a greater propensity to purchase green products as their environmental consciousness increases, in contrast to their more price-sensitive counterparts [45]. Leveraging these findings, this study advances H5 by examining the negative influence of price sensitivity on behavioral intentions.

This study examines Taiwanese consumers’ intentions to purchase upcycled food products using the VAB model as a conceptual framework, integrating “product knowledge”, “perceived green quality”, and “price sensitivity” as focal variables. We propose the following hypotheses:

**H1a.**  *consumers’ green consumption values positively influence their attitudes towards upcycled food.*

**H1b.**  *consumers’ green consumption values positively influence anticipated guilt.*

**H2a.**  *consumers’ attitudes towards upgraded recycled foods positively influence their behavioral intentions.*

**H2b.**  *consumers’ anticipated guilt positively influences their behavioral intentions.*

**H3.**  *product knowledge about upcycled food positively influences behavioral intention.*

**H4.**  *the green perceived quality of upcycled food positively impacts behavioral intentions.*

**H5.**  *the price sensitivity of consumers negatively affects their behavioral intentions.*

Through a comprehensive analysis of the impact of these factors on consumer purchasing intentions, strategic recommendations are proposed to foster the market expansion of upcycled foods, thereby aiding in climate change mitigation and enhancing food security.

## 2. Research Methodology

### 2.1. Research Framework

**Figure 1:**
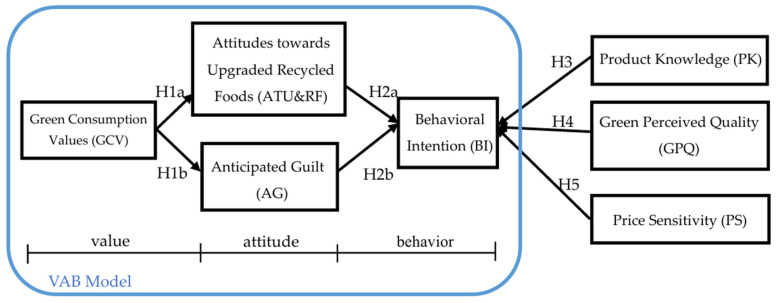
Research framework diagram.

By synthesizing the literature discussed above, this study focused on the VAB model by integrating three research variables: “Product Knowledge”, “Green Perceived Quality”, and “Price Sensitivity”, as depicted in Figure 1.

### 2.2. Questionnaire Development

The questionnaire design comprises seven parts. The first segment focuses on product knowledge derived from Sun and Wang [46] and comprises three questions. The next section addresses green consumer values by adapting the study by Do Paco et al. [47] to six questions. The third part explored attitudes towards upcycled food, drawing from modifications of the research by Kamalanon et al. [48] and involving four questions. The subsequent section delves into anticipated guilt based on modifications of the study by Attiq et al. [49] with four questions. The following sections cover green perceived quality, price sensitivity, and behavioral intentions, adapting the studies by Riva et al. [40], Ogiemwonyi [42], and Rausch and Kopplin [50]. The eighth part gathered demographic data using a 7-point Likert scale for all the questions.

To ensure the clarity and accuracy of the questions and prevent misinterpretation, an expert validity review was conducted. Nine experts, including educational scholars and food industry professionals, each with more than a decade of experience, were invited to review and modify the questionnaire for precision and appropriateness. Their input was consolidated to refine the questionnaire. A pilot test with 64 questionnaires validated the reliability of the items through item and reliability analysis.

Following this rigorous process, the final questionnaire design was established and responses were scrutinized to eliminate incomplete or inconsistent data. Cronbach’s alpha values for each construct ranged from 0.843 to 0.925, indicating strong reliability.

### 2.3. Sample and Data Collection

In light of technological advancements and the prevalence of the Internet, researchers have shifted towards online questionnaire dissemination for data collection. While online surveys may exhibit lower response rates, strategies, such as advance notifications and concise surveys, can improve participation. Online questionnaires offer several advantages in terms of data integrity and resource efficiency. This study employed convenience sampling and distributed questionnaires through various online platforms and emphasized the participants’ privacy and anonymity. Statistical analyses were performed using structural equation modeling, with a sample size of 320 effective questionnaires collected from 335 distributed questionnaires, meeting the criteria for robust analysis.

### 2.4. Sampling and Data Acquisition

As digital advancements and the ubiquity of the Internet reshape our communication methods, an increasing number of social science researchers are transitioning from traditional paper-based surveys to the virtual dissemination of questionnaires via online platforms and social media networks. This shift not only facilitates research data collection but also aligns with contemporary communication trends. Sammut et al. [51] highlight that despite the generally lower response rates associated with online questionnaires, proactive measures, such as preemptive email notifications or the development of concise, 10 min surveys, can significantly enhance participation rates. Furthermore, digital questionnaires offer several advantages over their paper counterparts, including improved data integrity, resource efficiency, and the ability to garner more thorough responses.

In this digital arena, researchers have leveraged various dissemination channels, including social networks such as Facebook, Instagram, Line, and personal communities, to circulate their questionnaires. Adhering to ethical research standards, this study transparently communicated its objectives to the participants and guaranteed anonymity through the survey’s online portal, thus ensuring a comfortable environment for respondents free from privacy concerns.

### 2.5. Methods of Data Analysis

This study employed quantitative research methods and utilized IBM SPSS Statistics 27 and AMOS 28 for the data analysis. Statistical techniques included descriptive statistics, reliability and validity analyses, and SEM using maximum likelihood estimation to explore causal relationships and model fit. These methods were used to validate the research hypotheses outlined in this study.

## 3. Analysis and Results

### 3.1. Demographic Analysis

Given the specific needs of this study and the prerequisites for hypothesis testing, we employed SEM to analyze the collected data. Wu [52] posited that the optimal sample size for SEM is contingent upon the ratio of the number of items, recommending a range of 10:1 to 15:1. Consequently, with the 31 items presented in this investigation, the ideal sample size was projected to be between 310 and 465 respondents. Between February and June 2024, 335 responses were gathered using an official questionnaire. After excluding 15 invalid submissions, 320 valid questionnaires were obtained. Table 1 presents the demographic characteristics of the sample population.

### 3.2. Measurement Model: Reliability and Validity

This study employed a two-stage analysis method, with the first stage being confirmatory factor analysis (CFA) and the second stage being the analysis of the overall model fit. CFA is part of the SEM analysis used to assess the relationship between observed variables and latent factors, namely, whether the latent variables can truly be represented by the observed variables. Generally, CFA can be used to evaluate psychological measurements, construct validity, test method effectiveness, and examine model group invariance. As this research incorporated questionnaires developed by other researchers, it is necessary to use CFA to verify whether the measurement tool is appropriate for the study population.

This study consists of seven dimensions: “Product Knowledge”, “Green Consumption Values”, “Attitudes towards Upcycled Food”, “Anticipated Guilt”, “Green Perceived Quality”, “Price Sensitivity”, and “Behavioral Intentions”. Confirmatory factor analysis was conducted individually for each dimension. First, items with a factor loading of less than 0.4 were eliminated based on the outcomes, and confirmatory factor analysis was repeated to assess the root mean square error of approximation (RMSEA) of the sub-dimensions. If it is greater than 0.08, it indicates that it does not meet the fit criteria. Thus, based on the principle of deleting items according to the modification index (MI) value, the model was repeatedly modified until the RMSEA of the dimension was less than 0.08, or the subdimension became a saturated model.

After confirming the dimensions of the scale, the composite reliability (CR) and convergent validity of each dimension were tested immediately. The CR value represents the combination of all the reliability of the measurement variables, and it is a ratio ranging from zero to one. The higher the CR value, the higher the proportion of the “true variance out of the total variance”, which indicates higher internal consistency. Fornell and Larcker [53] suggested that the CR value of the latent variables should be greater than 0.60. The convergent validity of latent variables is best represented by the average variance extracted (AVE). Both Fornell and Larcker [53] and Bagozzi and Yi [54] recommend that the AVE of latent variables should preferably exceed 0.50. In this study, the CR values of the scale dimensions ranged from 0.906 to 0.947, indicating that the scale had good internal consistency. The AVE values ranged from 0.668 to 0.816, exceeding the recommended value of 0.50, indicating that the scale had good convergent validity. The standardized regression weights of all items ranged from 0.645 to 0.922, and the t-values were greater than 1.96; therefore, all were significant. The factor loadings, dimension CR values, and AVE values are presented in Table 2. The content of the table shows that the dimensions of this questionnaire met the requirements of convergent validity; hence, the measurement model had good internal quality.

SEM discriminant validity analysis involves measuring two different concepts and conducting a relevance analysis of the results. If the degree of correlation is very low, it indicates discriminant validity between the two concepts. According to Hair et al. [55], the correlation coefficient between two different concepts should be less than the square root of the AVE for each concept. Table 3 presents a comparison of all construct correlation coefficients and the square root of the AVE in this study. The square root values of the AVE for each construct were greater than the correlation coefficients between the constructs, meeting the standard recommended by Hair et al. [55], which shows discriminant validity among the constructs in this study. Based on the evaluation results of the measurement model, it can be concluded that the measurement model used in this study had good internal and external qualities.

### 3.3. Model Fit Test

Table 4 presents the results of the fit index analysis. This study employed the maximum likelihood (ML) estimation method to construct a structural model to test the hypothesized relationships of the proposed model. The relevant indices are as shown in Table 4: the chi-square to degrees of freedom ratio (x^2^/*df*) = 3.145, root mean square residual (RMR) = 0.043, root mean square error of approximation (RMSEA) = 0.079, adjusted goodness of fit index (AGFI) = 0.814, normed fit index (NFI) = 0.904, comparative fit index (CFI) = 0.911, and incremental fit index (IFI) = 0.912, all of which met the standards, indicating that the overall model of this study demonstrated a good fit.

### 3.4. Overall Model Path Analysis

**Figure 2:**
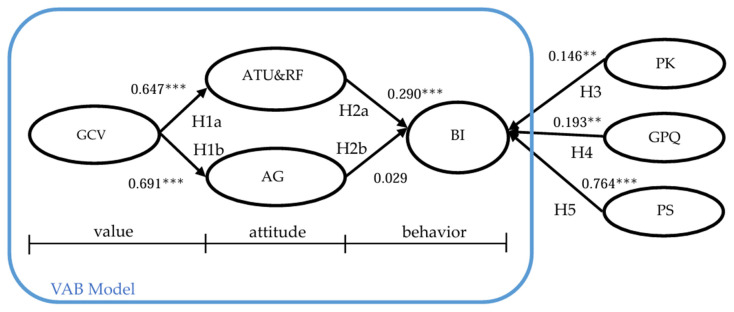
Structural equation modeling diagram. Note: ** *p* < 0.01; *** *p* < 0.001.

This study employed SEM to examine the relationships between various variables and conducted a detailed analysis within the proposed theoretical framework. The structural model analysis diagram is shown in Figure 2.

H1a and H1b indicate that green consumer values have a significant positive impact on attitudes towards upcycled food (β = 0.647, *p* < 0.001) and anticipated guilt (β = 0.691, *p* < 0.001), confirming the role of individual values in shaping attitudes, as posited by VAB theory.

H2a, and H3–H4 describe how attitudes towards upcycled food (β = 0.290, *p* < 0.001), product knowledge (β = 0.146, *p* < 0.001), and green perceived quality (β = 0.193, *p* < 0.001) significantly influence behavioral intentions, highlighting the multifaceted motivational basis for consuming upcycled food products.

However, H2b shows that anticipated guilt has a positive but not significant effect on behavioral intentions (β = 0.029, *p* = 0.629); thus, the hypothesis is not supported. This suggests that more research is needed to understand the role and impact of this emotional factor in decision making.

H5 shows that price sensitivity significantly and positively affects consumers’ behavioral intentions (β = 0.764, *p* < 0.001), contrary to the original hypothesis that it negatively affects behavioral intentions; thus, the hypothesis is not supported. This indicates that consumers may place more emphasis on price than expected and that price sensitivity promotes purchase intentions.

Therefore, it can be known that H1a, H1b, H2a, H3, and H4 are all valid and significant, while H2b and H5 are not. Table 5 presents the path analysis and hypothesis testing results of this study.

## 4. Discussion

This study explores the factors influencing Taiwanese consumers’ decisions to purchase upgraded recycled food through the VAB theory. The findings provide substantive insights into consumer behavior dynamics by employing structural equation modeling analysis. First, it was discerned that consumers’ green consumption values notably and positively influenced their attitudes towards upgraded recycled food, alongside an augmented anticipation of guilt. This observation suggests that values underpinned by environmental and social responsibilities not only foster a positive disposition towards sustainable food options but also amplify guilt anticipation associated with the potential selection of non-eco-friendly choices. These insights are consistent with the findings of Lu et al. [29] and Roh et al. [56], who similarly underscore the pivotal role of values in steering consumer behavior towards environmentally sustainable choices.

Moreover, this study corroborates the notion that a favorable attitude towards innovative and sustainable food solutions markedly influences behavioral intentions towards such products, echoing the findings of Chatterjee et al. [33] and Jung et al. [57] in the realms of green products and sustainable fashion. This indicates that positive perceptions can significantly incentivize consumers to prefer upgraded and reinvented food products. In contrast, anticipated guilt did not exhibit a statistically significant impact on behavioral intentions, diverging from the inference drawn by Jiang et al. [34] regarding its positive correlation with intention to adopt low-carbon consumption behaviors. This discrepancy could stem from the heterogeneity in individual sensitivity towards anticipated guilt and the perceived ramifications of certain actions, suggesting that not all individuals respond uniformly to emotional cues. Previous investigations by Yang et al. [58], Chen [59], and Lu et al. [29] collectively indicate that emotional responses play a crucial role in behavior prediction. Hence, this divergence warrants a more nuanced exploration of how different psychological, cognitive, and situational factors mediate the relationship between emotional antecedents and consumer behavioral intentions.

Additionally, the significance of product knowledge on behavioral intentions was affirmed, highlighting that a deeper understanding of a product fosters positive purchase intentions and actions. This finding corroborates Ayub and Kusumadewi [37] and Liu et al. [60] and highlights the critical role of information and education in molding consumer decisions [61]. Similarly, the perceived green quality of products was found to significantly impact consumer behavioral intentions positively, resonating with Riva et al. [40] and Vuong and Nguyen [41], further illustrating consumers’ inclination towards products that not only satisfy their environmental values but also embody their social and environmental responsibilities [62,63].

Notably, our findings on price sensitivity diverge from the extant literature, indicating a positive correlation with behavioral intentions, contrary to the anticipated negative relationship. Several studies [64,65] have suggested that while positive intentions may prevail, elevated price sensitivity can deter actual purchase behavior. However, Ogiemwonyi [42] unveiled a distinctive dichotomy within green consumer behavior, in which a subset of consumers were willing to pay for sustainable products and services. This delineates a nuanced consumer segment for whom quality supersedes price sensitivity concerning environmental goods, thus diluting the assumed negative impact of price on sustainable purchase behaviors.

Our research found that green consumption values play a significant role in Taiwanese consumers’ attitudes and behavioral intentions towards upcycled foods, consistent with the findings from other countries. For example, Turkish consumers are more interested in purchasing such products when they perceive them as helping solve food waste issues [66]. However, differences in cultural background and values lead to differences between countries. For instance, Dutch and Swedish consumers emphasize moral self-reward and environmental benefits more [67,68], whereas in the United States, esthetic and emotional values are given more importance [69]. Additionally, studies in these countries have highlighted the influence of consumer perceptions of product quality, nutritional value, and environmental benefits on purchase intentions. These comparisons help broaden our understanding of global consumer motivations for upcycled foods and underscore the important role of cultural differences in consumer behavior.

In summary, this investigation meticulously explores the interplay between green consumer values, attitudes towards sustainably upgraded food, and behavioral intentions through the lens of VAB theory. It uncovers the multifaceted nature of emotional underpinnings, emphasizes the cruciality of informed decision making, and delineates the perceived quality of green products as seminal influencers of consumer purchase inclinations. Together, these findings not only enrich our understanding of consumer behavior in the sustainable food sector but also signal pivotal considerations for marketers and policymakers aiming to foster a more ecological consumption landscape.

## 5. Conclusions and Recommendations

### 5.1. Research Conclusions

This study investigates the influence of value–attitude–behavior (VAB) theory, incorporating green perceived quality, product knowledge, and price sensitivity, on Taiwanese consumers’ behavioral intentions towards upgraded reclaimed food products. The findings highlight the significant role of green consumption values in forming positive attitudes towards these products. Despite the expected impact, anticipated guilt was not significantly correlated with behavioral intention, indicating complex emotional influences on behavior. Perceived green quality and product knowledge emerged as significant predictors of behavioral intention, whereas price sensitivity surprisingly showed a positive influence, suggesting that consumers value intrinsic quality and ideological appeal over price.

### 5.2. Managerial Implications

This study provides strategic insights into enhancing consumer engagement with upcycled food products.

1. Marketing aligned with green values: emphasizing the environmental benefits of upcycled foods in marketing to foster consumer acceptance.
2. Enhancing product knowledge: educate consumers through workshops and demonstrations to increase purchase intentions.
3. Boosting green quality perception: obtain environmental certifications and promote eco-friendliness to build brand trust.
4. Governmental support: implement subsidies, regulations, and promotional initiatives to create a supportive market for upcycled foods.

### 5.3. Research Limitations and Future Research Directions

While this study offers valuable insights, its limitations include a lack of cultural diversity and a detailed demographic analysis. Future research should explore cross-cultural studies and demographic variables to enhance the generalizability of the findings. Additionally, alternative theoretical frameworks and variables, such as information asymmetry and neophobia, should be examined. Understanding the moderating role of emotional responses could further elucidate consumer behavior towards upcycled food.

This study only discussed Taiwanese individuals; however, the consumption of different foods is highly correlated with culture. Therefore, future research should delve deeper into cultural differences (i.e., differences between linguistic regions or countries) to examine the universality and applicability of the study results.

Convenience sampling was employed in our study. However, according to Andrade [70] and Emerson [71], convenience sampling may lack generalizability, potentially restricting the broad applicability of our findings. Future research could utilize different sampling methods (e.g., stratified sampling) to ensure the representativeness of each subgroup in the sample based on specific attributes, thereby enhancing the generalizability of the overall results.

In conclusion, this study advances the discourse on consumer behavior within the VAB framework, providing a foundation for future research and managerial practices aimed at promoting sustainable consumption patterns.

## Figures and Tables

**Table 1:** Demographic analysis.

*n* = 320	Item	Population	Percentage (%)
Gender	Male	158	50.6
	Female	162	49.4
Age	20 years and below	24	7.5
	21–30 years	69	21.6
	31–40 years old	92	28.7
	41–50 years old	72	22.5
	51–60 years	47	14.7
	60 years and above	16	5.0
Education Level	High school/vocational or below	47	14.7
	College/university	207	64.7
	Master’s or above	66	20.6
Personal monthly income	Less than NTD 20,000 (USD 660) (inclusive)	49	15.3
	NTD 20,001–40,000 (USD 660–1320)	112	35.0
	NTD 40,001–60,000 (USD 1320–1980)	92	28.7
	NTD 60,001–80,000 (USD 1980–2640)	30	9.4
	Above NTD 80,001 (USD 2640)	37	11.6

**Table 2:** Results related to factor loading, reliability, and validity.

Variables/Items	Mean	Standard Deviation	Standardized Factor Loading	AVE	CR
Product Knowledge (PK)	3.93	1.557		0.762	0.906
1. I have a deep understanding of the content, processes, and applications of food upgrading and remanufacturing.	3.96	1.722	0.893 ***		
2. I often come across upgrading and remanufacturing food during my daily shopping experience.	4.07	1.783	0.857 ***		
3. I acquired information about upgrading and remanufacturing food by reading academic journals, professional reports, or related research.	3.75	1.846	0.869 ***		
Green Consumption Values (GCV)	5.22	1.113		0.668	0.923
4. Choosing products that are environmentally friendly and not harmful is an important consideration in my consumption behavior.	5.29	1.335	0.845 ***		
5. When making consumption decisions, I often consider the impact of my actions on the environment.	4.98	1.456	0.870 ***		
6. My concern for environmental issues affects my purchasing behavior.	5.15	1.399	0.857 ***		
7. I believe that the excessive consumption of Earth’s resources has adverse effects on both the self and society.	5.80	1.262	0.645 ***		
8. I see myself as a consumer who actively supports environmental protection.	4.92	1.402	0.863 ***		
9. I am willing to accept some inconveniences in life because of the environmental principles.	5.19	1.319	0.802 ***		
Attitude Towards Upgrading and Remanufacturing Food (ATU & RF)	5.19	1.138		0.734	0.917
10. This study supports the concept of purchasing upgrades and remanufacturing food.	5.12	1.314	0.875 ***		
11. I hold a positive attitude towards purchasing upgrades and remanufacturing food.	5.09	1.309	0.913 ***		
12. When choosing products, I place great importance on their contribution to environmental protection.	5.24	1.349	0.760 ***		
13. I believe that purchasing upgraded and remanufactured food has a positive significance for environmental and resource conservation.	5.33	1.357	0.871 ***		
Anticipated Guilt (AG)	5.41	1.225		0.816	0.947
14. I feel a moral burden when I realize that I am wasting food.	5.58	1.325	0.891 ***		
15. The negative impact of food waste on the economy and society makes me feel guilty.	5.33	1.414	0.913 ***		
16. The negative environmental impact of food waste makes me feel ashamed.	5.18	1.374	0.922 ***		
17. Knowing that food waste can lead to hunger issues makes me feel guilty and motivates me to take actions to reduce waste.	5.53	1.308	0.887 ***		
Green Perceived Quality (GPQ)	5.51	0.966		0.670	0.910
18. I prefer green products with high environmental quality ratings.	5.16	1.302	0.820 ***		
19. I tend to choose products that are stable and reliable in performance, and have environmental certifications.	5.48	1.182	0.869 ***		
20. I prefer products with good green brand reputation.	5.28	1.302	0.890 ***		
21. For branded products that I have had a good user experience, I have a higher trust in quality.	5.88	1.052	0.752 ***		
22. I believe that product brands are recommended by reliable sources.	5.75	1.044	0.750 ***		
Price Sensitivity (PS)	4.84	1.154		0.706	0.923
23. I am willing to pay a higher price for high-quality food upgrades and remanufacturing.	4.68	1.511	0.827 ***		
24. I believe that the price of upgrading and remanufacturing food should reflect its benefits.	5.18	1.289	0.780 ***		
25. I believe that the price for upgrading and remanufacturing food is reasonable.	4.62	1.373	0.850 ***		
26. I believe that the price of upgrading and remanufacturing food is commensurate with its value.	4.86	1.298	0.892 ***		
27. From an economic perspective, I believe that choosing to upgrade and remanufacture food is a wiser choice.	4.87	1.401	0.847 ***		
Behavioral Intentions (BI)	4.84	1.158		0.740	0.919
28. I will consider purchasing upgrades and remanufacturing food.	4.66	1.390	0.870 ***		
29. I plan to upgrade and remanufacture traditional food in the future.	4.48	1.403	0.847 ***		
30. I think it is possible to purchase upgrades and remanufacture food in the future.	5.07	1.294	0.887 ***		
31. When I see upgrading and remanufacturing food online or in physical stores, I consider purchasing it in appropriate circumstances.	5.14	1.303	0.835 ***		

Note 1: CR = composite reliability; AVE = average variance extracted. Note 2: *** *p* < 0.001.

**Table 3:** Discriminant validity rest.

	1.	2.	3.	4.	5.	6.	7.
1. PK	**0.873**						
2. GCV	0.386 **	**0.817**					
3. ATU & RF	0.386 **	0.653 **	**0.857**				
4. AG	0.286 **	0.642 **	0.563 **	**0.903**			
5. GPQ	0.280 **	0.749 **	0.661 **	0.655 **	**0.818**		
6. PS	0.422 **	0.648 **	0.723 **	0.547 **	0.690 **	**0.840**	
7. BI	0.428 **	0.571 **	0.696 **	0.514 **	0.652 **	0.812 **	**0.860**

Note 1: The values in bold font are the square roots of the AVE; non-diagonal numbers represent the correlation coefficients of each dimension. Note 2: PK = product knowledge; GCV = green consumption values; ATU & RF = attitude towards upgrading and remanufacturing food; AG = anticipated guilt; GPQ = green perceived quality; PS = price sensitivity; BI = behavioral intentions. Note 3: ** *p* < 0.01.

**Table 4:** Analysis of fit indices.

Statistic		Recommended Value	Obtained Value	Meets Standard
Absolute Fit Indices	χ^2^/*df*	<5	3.145	Yes
	RMR	<0.05	0.043	Yes
	RMSEA	≤0.05 (marginal fit)	0.079	Good fit
		0.05–0.08 (good fit)		
		0.08–0.10 (moderate fit)		
		>0.10 (poor fit)		
Incremental Fit Indices	AGFI	>0.8	0.814	Yes
	NFI	>0.9	0.904	Yes
	CFI	>0.9	0.911	Yes
	IFI	>0.9	0.912	Yes

Note: root mean square residual (RMR), root mean square error of approximation (RMSEA), adjusted goodness of fit index (AGFI), normed fit index (NFI), comparative fit index (CFI), incremental fit index (IFI).

**Table 5:** Results of the path analysis and confirmation of hypotheses.

Hypothesized Paths	Unstandardized Coefficient	S.E.	C.R.	Standardized Coefficients	β	*p*	Verification Results
H1a: GCV→ATU & RF	0.847	0.090	9.346	0.647	0.653	***	Supported
H1b: GCV→AG	0.955	0.091	10.507	0.691	0.642	***	Supported
H2a: ATU & RF→BI	0.428	0.108	3.967	0.290	0.696	***	Supported
H2b: AG→BI	0.041	0.084	0.483	0.029	0.514	0.629	Unsupported
H3: PK→BI	0.284	0.103	2.746	0.146	0.428	**	Supported
H4: GPQ→BI	0.374	0.141	2.660	0.193	0.141	**	Supported
H5: PS→BI	1.480	0.219	6.754	0.764	0.812	***	Unsupported

Note 1: PK = product knowledge, GCV = green consumption values, ATU & RF = attitude towards upgrading and remanufacturing food, AG = anticipated guilt, GPQ = green perceived quality, PS = price sensitivity, BI = behavioral intentions. Note 2: *** *p*-value < 0.001; ** *p*-value < 0.05.

## Data Availability

The data presented in this study are available on request from the corresponding author due privacy.
